# Atypical Neural Responses of Cognitive Flexibility in Parents of Children With Autism Spectrum Disorder

**DOI:** 10.3389/fnins.2021.747273

**Published:** 2021-12-17

**Authors:** Xin Cheng, Yu Li, Xiwen Cui, Hong Cheng, Chunyan Li, Linyan Fu, Jiying Jiang, Zhenyu Hu, Xiaoyan Ke

**Affiliations:** ^1^The Child Mental Health Research Center, Nanjing Brain Hospital Affiliated to Nanjing Medical University, Nanjing, China; ^2^Department of Child Psychiatry, Ningbo Kangning Hospital, Ningbo, China; ^3^Physical Diagnostic Department, Nanjing Brain Hospital Affiliated to Nanjing Medical University, Nanjing, China

**Keywords:** autism spectrum disorder, first-degree relatives, cognitive flexibility, N2, P3

## Abstract

Impaired cognitive flexibility has been repeatedly demonstrated in autism spectrum disorder (ASD). There is strong evidence for genetic involvement in ASD. First-degree relatives of individuals with ASD may show mild deficits in cognitive inflexibility. The present study investigated cognitive flexibility and its neuroelectrophysiological mechanisms in first-degree relatives of individuals with ASD to assess its potential familiality. Forty-five biological parents of individuals/children with ASD (pASD) and thirty-one biological parents of typically developing individuals/children (pTD), matched by gender, age, and IQ, were enrolled. The broad autism phenotype questionnaire (BAPQ) and cognitive flexibility inventory (CFI) were used to quantitatively assess autistic traits and cognitive flexibility in daily life, respectively. The task-switching paradigm was used to evaluate the behavioral flexibility in a structured assessment situation. Event-related potentials (ERPs) induced by this paradigm were also collected. Results showed that compared with the pTD group, the pASD group had lower CFI scores (*t* = −2.756, *p* < 0.01), while both groups showed an equivalent “switch cost” in the task-switching task (*p* > 0.05). Compared with the pTD group, the pASD group induced greater N2 amplitude at F3, F4, Fz, and C4 (*F* = 3.223, *p* < 0.05), while P3 amplitude and latency did not differ between the two groups. In addition, there was a significant negative correlation between the CFI total scores and BAPQ total scores in the pASD group (*r* = −0.734, *p* < 0.01). After controlling for age and IQ, the N2 amplitude in the frontal lobe of pASD was negatively correlated with the CFI total scores under the repetition sequence (*r* = −0.304, *p* = 0.053). These results indicated that pASD had deficit in cognitive flexibility at the self-reported and neurological levels. The cognitive flexibility difficulties of parents of children with ASD were related to autistic traits. These findings support that cognitive flexibility is most likely a neurocognitive endophenotype of ASD, which is worthy of further investigation.

## Introduction

Autism spectrum disorder (ASD) is a neurodevelopmental disorder characterized by persistent difficulties in social communication and interaction, as well as restricted, repetitive patterns of behavior. A number of studies have found that unaffected family members of ASD individuals share some behavioral and cognitive traits with probands, but to a lesser extent, known as the broader autism phenotype (BAP) (Piven et al., 1997), which indicates that the core autistic traits can be passed from generation to generation. However, even with heritability estimated as high as 74∼93% (Tick et al., 2016), our understanding of the underlying pathophysiological mechanism and their relationship to autistic characteristics remains unclear. This is partly due to the lack of well-established biomarkers associated with core clinical features. Therefore, characterizing the brain profiles of unaffected first-degree relatives of ASD individuals may be helpful to understand the characteristic patterns of intergenerational inheritance, identify endophenotypes of ASD, and bridge etiological processes and clinical phenotypes.

Cognitive flexibility is one of the core executive functions, referring to the ability to adjust behaviors appropriately to environmental changes (Dajani and Uddin, 2015). It is very important for goal-oriented and adaptive behaviors. In the laboratory environment, cognitive flexibility is usually measured using the Wisconsin Card Sorting Test (WCST), task switching and set-shifting paradigms. Impaired cognitive flexibility in ASD individuals of different ages has been repeatedly demonstrated (D’Cruz et al., 2013; Rosenthal et al., 2013; Van Eylen et al., 2017; Johnston et al., 2019; Schmitt et al., 2019; Xie et al., 2020). Also, multiple studies have also shown that cognitive flexibility is closely related to stereotypical behaviors (Lopez et al., 2005; Miller et al., 2015; Faja and Nelson Darling, 2019; Iversen and Lewis, 2021). Thus, cognitive flexibility is regarded as one of the neurocognitive dimensions associated with the core clinical features of ASD, closely related to the underlying neurobiological processes.

Several studies have shown deficit in behavioral flexibility in unaffected first-degree relatives of individuals with ASD, suggesting that it may serve as neurocognitive traits linked to familiality (Hughes et al., 1999; Moazzen et al., 2015; Li et al., 2017; Schmitt et al., 2019; Shalani et al., 2019). However, some studies have reported that unaffected first-degree relatives of individuals with ASD retained intact cognitive flexibility (Wong et al., 2006; McLean et al., 2014; Rosa et al., 2017). Mixed findings may result from differences in subject characteristics, such as age and sample size. For example, a study demonstrated significant differences between 124 parents and siblings of autistic children and 124 parents of typically developing children in WCST (Moazzen et al., 2015), whereas another study found 22 unaffected siblings of ASD individuals performed similarly to control participants in WCST (Rosa et al., 2017). In addition, there are inconsistencies between measures. For example, a study showed neither parents nor siblings of individuals with ASD displayed significant difficulties in set-shifting (Wong et al., 2006).

Previous behavioral findings suggested that there may be abnormalities in the executive control networks in ASD, especially those involving cognitive flexibility. It is reported that the lateral frontal parietal network (L-FPN) and the middle cingulate gyrus-insular network (M-CIN) play a central role in supporting executive function and cognitive flexibility (Uddin, 2021). The literature has showed aberrant patterns in these brain regions related to cognitive flexibility in ASD, including frontal and parietal lobes (Shafritz et al., 2008; Yerys et al., 2015; Lynch et al., 2017). However, little is known about more precise cognitive processing and the underlying pathobiological mechanism of cognitive flexibility in ASD. Event-related potentials (ERPs) are time-locked measures of event-related electrical activity in the brain, providing neural processes underlying specific cognitive and behavioral responses. The N2 is a late negative fluctuation observed approximately 200 ms after a stimulus onset (Cremone-Caira et al., 2020). The P3, a late positive waveform that occurs at a latency of approximately 300 ms after a stimulus onset, is known to reflect executive and attentional function, working memory, event categorization, and attentional resource allocation (Polich, 2007). Previous studies in healthy people have shown that N2 and P3 are frequently observed during task switching (Karayanidis et al., 2010; Kopp et al., 2020). It is well known that P3 shows a maximum amplitude in the parietal lobe, and the inferior and posterior parietal regions are associated with P3 amplitude modulation during task switching (Petruo et al., 2019). The N2 is strongly related to frontocentral regions (Kopp et al., 2020) and it reflects attentional control and inhibition, and its amplitude varies with changes in conflict and the need for cognitive control. As far as we know, only a few studies have reported changes in the ERPs of cognitive flexibility in ASD. Moreover, differences in ERP patterns between ASD and typically developing individuals vary depending on task types and developmental levels. For example, a study found that when ASD adolescents over 16 years had larger N2 during a Go/NoGo task, compared with the control group, there was no significant difference in P3 (Høyland et al., 2017). Another study reported that there was no significant difference in P3 between the ASD group and the control group during task switching (Hoofs et al., 2018).

As described above, several studies have reported deficit in behavioral flexibility in first-degree relatives of autistic children, although findings were inconsistent. What’s more, neuroimaging studies have shown that first-degree relatives of autistic children had abnormal activation patterns in the frontal lobes, cingulate gyrus and parietal lobe, which were core brain areas supporting cognitive flexibility (Spencer et al., 2012; Dajani and Uddin, 2015; Moseley et al., 2015; Mehdizadehfar et al., 2020; Peng et al., 2020). Therefore, it is reasonable to assume that cognitive flexibility in unaffected first-degree relatives of autistic individuals may be impaired and manifested at the neurological level. However, to the best of our knowledge, no studies have specifically explored the neuroelectrophysiological mechanism of cognitive flexibility in first-degree relatives of ASD probands.

Therefore, the present study aimed to compare and analyze the differences in cognitive flexibility between parents of children with ASD and typically developing children and use the ERPs technique to accurately analyze the neural activity changes in parents of children with ASD during task-switching paradigm. We also investigated the relationship between ERPs (N2, P3) and cognitive flexibility to determine whether such neuroelectrophysiological differences might affect cognitive flexibility. This study also assessed the extent to which cognitive flexibility deficits covaried with subclinical autistic traits in unaffected relatives to better understand the intergenerational transmission of behavioral traits associated with ASD. Based on previous findings, we hypothesized that the cognitive flexibility in the parents of individuals with ASD (pASD) group was worse than that in the control group. We also predicted higher ERPs amplitudes generated in the pASD group, reflecting increased efforts at the task switching process. Finally, we expected to find a positive covariant relationship between cognitive flexibility deficits and subclinical autistic traits.

## Materials and Methods

### Participants

Parents of children with ASD were recruited in the outpatient and rehabilitation department of Nanjing Brain Hospital and the control group were recruited through advertisements. Biological parents of 31 children with ASD (30 boys; mean age: 5.38 ± 2.11 years), diagnosed by two senior child psychiatrists according to the diagnostic criteria for ASD in the *Diagnostic and Statistical Manual of Mental Disorders*, Fifth Edition (DSM-5), and 23 typically developing children (15 boys; mean age: 6.48 ± 2.73 years) participated in the study. In total, forty-five biological parents (20 fathers, 25 mothers) of children with ASD (pASD) participated in the study. Thirty-one biological parents (15 fathers, 16 mothers) of typically developing children (pTD) were recruited to serve as the control group. It is worth noting that pTD did not give birth to children with neurodevelopmental disorders, such as ASD. All participants were right-handed and had normal or corrected vision. All participants had an IQ score greater than 80. The IQ was estimated using the short form of the Wechsler Adult Intelligence Scale. In addition, participants who had a history of psychiatric illness, serious physical disease, taking psychotropic drugs in the past month or EEG examination contraindication were excluded.

This study has been reviewed and approved by the Medical Ethics Committee of Nanjing Brain Hospital Affiliated to Nanjing Medical University (2020-KY104-01). According to the Declaration of Helsinki, after all participants were given informed consent to this study and signed informed consent, they first completed a series of questionnaires and assessments (see section “Assessment” for details), and then underwent an EEG recording while performing task-switching task in Nanjing Brain Hospital. Demographic information of participants was summarized in Table 1.

**TABLE 1:** Comparison of demographic characteristics between the two groups.

	pASD	pTD	Statistics	*p*
Male/female	20/25	15/16	χ^2^ (0.115)	0.735
Age, years	35.29 ± 3.89	36.84 ± 4.37	*t*(−1.622)	0.109
Intelligence quotient (IQ)	113.23 ± 11.67	113.68 ± 8.12	*t*(−0.176)	0.861
BAPQ total scores	92.99 ± 23.95	92.37 ± 13.56	*t*(0.143)	0.887
CFI total scores	72.38 ± 11.28	79.33 ± 9.78	*t*(−2.756)	0.007**

BAPQ, Broad Autism Phenotype Questionnaire; CFI, Cognitive Flexibility Inventory. **p < 0.01.

### Assessment

(1) Wechsler Abbreviated Scale of Intelligence (WASI) (Dumont et al., 2013): It was used to assess general intelligence in this study. It consisted of knowledge (I), similarity (S), mapping (PC), and block (BD). The short form yields an IQ score with a mean of 100 and a standard deviation of 15.
(2) Broad Autism Phenotype Questionnaire (BAPQ) (Hurley et al., 2007): It has a total of 36 items and is used to quantify the level of autistic traits in non-ASD people. It consists of three subscales, including aloof, rigid, and pragmatic language. The questionnaire is based on a 6-point Likert scale, which ranges from rarely (1) to always (6). Higher scores on the BAPQ indicate greater severity of autistic traits.
(3) Cognitive Flexibility Inventory (CFI) (Dennis and Wal, 2010): This is a 20-item, two-subscale self-reported questionnaire designed to assess aspects of cognitive flexibility that enables individuals to think adaptively rather than maladaptively when encountering stressful life events. The questionnaire is based on a 5-point Likert scale, which ranges from rarely (1) to always (5). Lower scores indicate worse cognitive flexibility. The Chinese version of the CFI has good reliability and validity (Wang et al., 2016). CFI has been used in parents of ASD individuals (Moradi et al., 2021).

### Experimental Tasks

**FIGURE 1:**
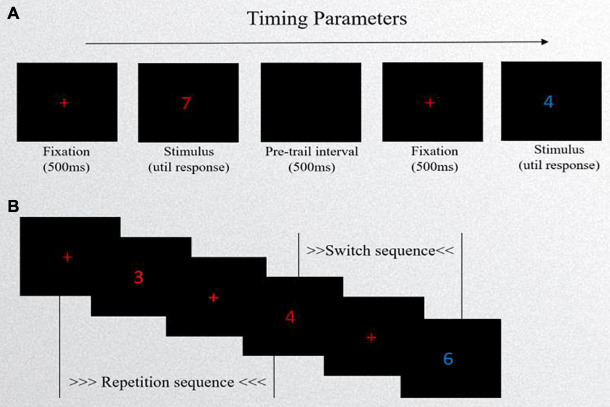
Schematic of task-switching task. **(A)** Experimental procedure: first, a red “+” fixation point occurred in the center of the computer screen, lasting for 500 ms, and then a target stimulus (a number in red or blue) was presented with no time limit. Lastly, the next trail appeared after a blank screen lasting 500 ms. **(B)** The presentation forms of stimuli. If the cue (the color of number) is the same as the previous cue, it is called a repetition sequence. If it is different from the previous one, it is called a switch sequence.

In this study, a task-switching paradigm was adopted (see Figure 1). The task was implemented in E-Prime 2.0 software. Participants were instructed to switch between two different types of tasks [odd–even (OE) vs. high–low (HL) task] based on external cues (color of digits).

#### Stimuli and Design

The stimuli were composed of the digits 1∼9, excluding 5, and each digit had two colors of red and blue. The number’s colors cued the tasks. The presentation forms of stimuli included repetition sequences and switch sequences. If the cue was the same as the previous cue, it was called a repetition sequence. If it differed from the previous one, it was called a switch sequence. The two types of sequences appeared randomly. In the task, repetition sequences accounted for 40% and switch sequences accounted for 60%. Participants had their performance evaluated on two task types: the odd–even task and the high–low task. The OE task required participants to classify the stimulus number as either “odd” or “even” when a red number appeared centered. The HL task required participants to classify the stimulus number as either “lower than 5” or “higher than 5” when a blue number appeared centered. Response box templates were created for the task so that the “F” button had a label of Odd/Low and the “J” button had a label of Even/High above the corresponding buttons. The experiment consisted of practice and formal sessions. The practice session consisted of pure-OE tasks (16 trials), pure-HL tasks (16 trials), and 32 mixed-condition trials with feedback. Then, they completed three mixed-condition blocks of 80 trials (without feedback) each, with a short break between the two blocks. Each number was presented in a random manner, appearing at the same frequency.

#### Experimental Procedure

First, a red “ + ” fixation point occurred in the center of the computer screen and lasted 500 ms, and then a number in red or blue was presented with no time limit. Lastly, the next trail appeared after a blank screen lasting 500 ms. The subjects were required to respond quickly and accurately when the stimulus presented. During the experiment, reaction time (RT) and errors were recorded. Data were cleaned of the first trials of each block, error trials, and trials from practice sessions. Next, trials with RT and error rates exceeding three standard deviations from the mean (considered per condition of each participant) were not included in the analysis.

### EEG Data Recording

EEG signals were continuously recorded while the subjects performed the task-switching task in a quiet room with dim lighting. The EEG signal was recorded with a 32-channel system produced by Brain Product, with the active electrodes situated on a standard cap according to the 10–20 system digitalized at 500 Hz. The reference electrode was placed at FCz, with a grounding electrode on AFz, and an electrode was placed under the right eye to record vertical electrooculography signals. Impedance of all electrodes were below 10 kΩ. The online filter was set at 0.016–100 Hz.

### Event-Related Potentials Analysis

Off-line EEG data were analyzed using EEGLAB v13.0.0 toolbox that operates within the MATLAB R2013b framework. Raw EEG signals were referenced to the average of the two earlobe electrodes and filtered between 0.5 and 30 Hz with a 50 Hz notch filter using a FIR filter. Trial epochs were extracted from −200 ms to +1000 ms with respect to target stimulus onset. Baseline correction was performed with the mean EEG signals 200 ms before the target stimulus. Artifacts such as eye movements and blinking were removed by independent component analysis (ICA). In addition, segments with amplitudes greater than ± 100 μV were eliminated. The ERPs for each individual were based on averaging the trials of the respective task condition after artifact correction. The ERPs were measured by the average amplitude method. The N2 (220–260 ms) and P3 (330–390 ms) at nine electrode points, including F3, F4, Fz, C3, C4, CZ, P3, P4, and Pz, were measured. The mean value in the F3, F4, and Fz electrodes was considered the mean amplitude within the frontal region.

### Statistical Analysis

The SPSS 23.0 software package was used for statistical analysis. Normality of the distributions was checked by the Shapiro–Wilk test. Categorical variables were investigated with χ^2^ tests, whereas normally distributed continuous variables, such as age, IQ, scale scores, were investigated with parametric test. Taking into account gender may affect cognitive flexibility (Zeestraten et al., 2017; Van’t Westeinde et al., 2020), we set it as a covariable. Accuracy and RT were analyzed by 2 (groups) × 2 (sequence types) repeated measures analysis of variance (RMANOVA). The Mann–Whitney U test was used to compare the difference in switch costs between the two groups (non-normally distributed data). A 2 (groups) × 2 (sequence types) × 9 (electrodes) RMANOVA was performed for the mean amplitude and latency of N2 and P3, respectively. Independent sample *t*-tests were used for post-tests, and Geisser-Greenhouse *P* value correction was used for multiple comparisons. Pearson correlation was used to investigate the correlations between CFI total scores and N2/P3 amplitude and BAPQ total scores. The test level was α = 0.05 (two-tailed).

## Results

### Demographic Characteristics in the Two Groups

There were no significant differences in sex, age, IQ, or BAPQ total scores between the two groups (*p* > 0.05). Parents of children with ASD had significantly lower CFI total scores than that of the controls (*p* < 0.01) (see Table 1).

### Behavioral Performances in the Task

#### Accuracy

The results of RMANOVA showed that the sequence types had a main effect [*F*(1, 73) = 12.398, *p* = 0.001], and the accuracy in the switch sequence was lower than that of repetition sequence (0.935 ± 0.10 vs. 0.947 ± 0.10). There was no main effect between groups, and the interaction between groups and sequence types was not significant (*p* > 0.05).

#### Reaction Time

The results of RMANOVA showed that the sequence types had a main effect [*F*(1, 73) = 10.487, *p* = 0.002], RT of the switch sequences was longer than that of the repetition sequences (1309.90 ± 37.11 vs. 1082.10 ± 23.67 ms). There was no main effect between groups, and the interaction between groups and sequence types was not significant (*p* > 0.05).

#### Switch Cost

There was no significant difference in switch cost (*Z* = −0.682, *p* > 0.05) between the two groups.

In conclusion, there were no significant differences in accuracy, response time or switch cost between the two groups.

### Event-Related Potential Data

#### N2

**FIGURE 2:**
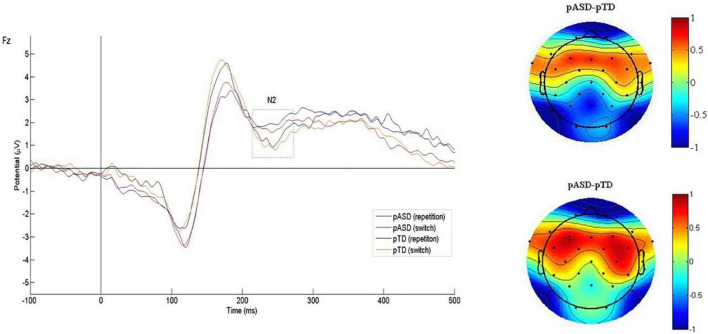
Grand average N2 waveforms and difference topographical maps (in μV). Grand average event-related N2 waveform measured at Fz for both conditions (switch/repetition) in the two groups (left). Group difference scalp topographical maps at Fz for both repetition (top right panel) and switch condition (bottom right panel).

The N2 waveform induced by the task is shown in Figure 2, and the mean N2 amplitude and latency of two groups are shown in Table 2.

**TABLE 2:** Mean amplitude and latency of N2 in the two groups.

	Amplitude (μV)		Latency (ms)	
	Repetition sequences	Switch sequences	Repetition sequences	Switch sequences
pASD	2.681 ± 0.278	2.674 ± 0.286	237.574 ± 1.705	236.883 ± 1.638
pTD	2.232 ± 0.334	1.925 ± 0.344	237.091 ± 2.055	237.644 ± 1.974

In the latency, RMANOVA results showed that the main effects of groups and sequence types were not statistically significant, and there was no interaction between groups, electrodes and sequence types (*p* > 0.05). The main effect of electrodes was significant [*F*(3.189, 8) = 3.344, *p* = 0.018].

**FIGURE 3:**
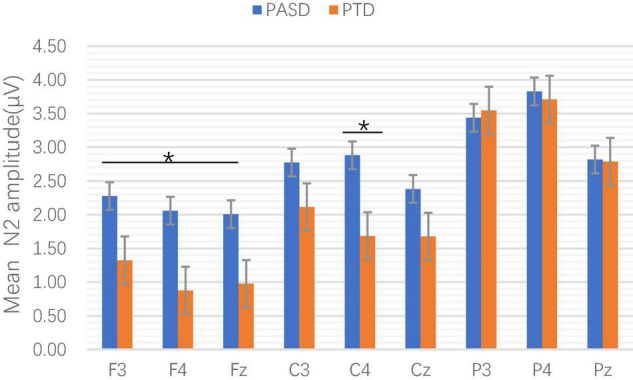
Comparison of mean N2 amplitude at each electrode point between the two groups. *p < 0.05.

In the amplitude, the main effects of electrodes, groups and sequence types were not significant, and there was no interaction between groups, electrodes and sequence types (*p* > 0.05). The interaction between electrodes and groups was statistically significant [*F*(2.510, 183.255) = 3.223, *p* = 0.031, η^2^*p* = 0.042]. Simple effect analysis showed that there were statistically significant differences in amplitude at F3 (*p* = 0.054, η^2^*p* = 0.050), F4 (*p* = 0.014, η^2^p = 0.079), Fz (*p* = 0.048, η^2^*p* = 0.052), and C4 (*p* = 0.019, η^2^*p* = 0.073) between the two groups (see Figure 3).

#### P3

**FIGURE 4:**
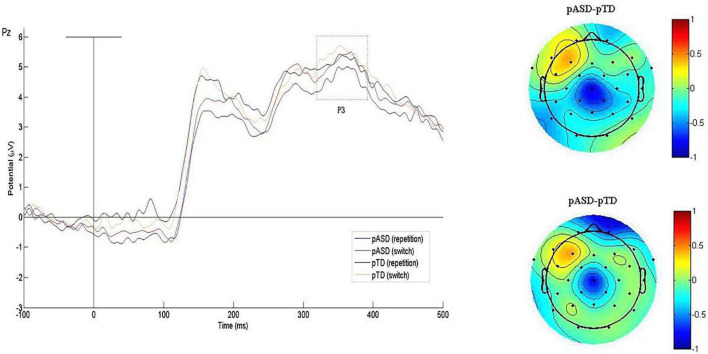
Grand average P3 waveforms and difference topographical maps (in μV). Grand average event-related P3 waveform measured at Pz for both conditions (switch/repetition) in the two groups (left). Group difference scalp topographical maps at Pz for both repetition (top right panel) and switch condition (bottom right panel).

The P3 waveform induced by the task is shown in Figure 4, and the mean P3 amplitude and latency of the two groups are shown in Table 3.

**TABLE 3:** Mean amplitude and latency of P3 in the two groups.

	Amplitude (μV)		Latency (ms)	
	Repetition sequence	Switch sequence	Repetition sequence	Switch sequence
pASD	3.683 ± 0.413	3.865 ± 0.377	355.162 ± 2.240	357.314 ± 2.233
pTD	3.956 ± 0.497	3.919 ± 0.455	357.237 ± 2.700	354.637 ± 2.690

In the latency, RMANOVA results showed that the main effects of groups and electrodes were not statistically significant, and there was no interaction between groups, electrodes and sequence types (*p* > 0.05). The main effect of sequence types was significant [*F*(1, 73) = 4.717, *p* = 0.033].

In the amplitude, the main effects of groups and sequence types were not significant, and there was no interaction between groups, electrodes and sequence types (*p* > 0.05). The interaction between electrodes and groups was also not significant. The main effect of electrodes was significant [*F*(1.964, 143.381) = 4.133, *p* = 0.019].

In summary, there were no significant differences in P3 latency and amplitude between the two groups.

### Brain-Behavior Correlation Analysis

The CFI total scores of pASD were negatively correlated with BAPQ total scores (*r* = −0.734, *p* < 0.001).

The mean amplitude of N2 in the frontal lobe was negatively correlated with the CFI total scores (*r* = −0.278, *p* = 0.016). After controlling for age and IQ, the mean N2 amplitude in the frontal lobe of ASD parents was negatively correlated with the CFI total scores under the repetition condition (*r* = −0.304, *p* = 0.053).

There was no correlation between the mean amplitude of P3 and CFI total scores (*p* > 0.05).

## Discussion

The current study evaluated cognitive flexibility in biological parents of individuals with ASD and typically developing individuals and further investigated the neuroelectrophysiological characteristics of cognitive inflexibility in the two groups. The results were partially consistent with our hypotheses. As expected, this study showed that parents of children with ASD had self-reported cognitive flexibility difficulties. In contrast, in a laboratory setting, their performance was comparable to the controls in task accuracy, response time, and switch costs. The present study is the first to investigate the neuroelectrophysiological characteristics of cognitive flexibility in pASD. We found that pASD induced significantly larger N2 amplitudes in the frontal lobe and right central region than the controls. However, there was no significant difference in P3 between the two groups. This study also found associations between self-reported cognitive flexibility difficulties and BAPQ total scores and N2 amplitude in the frontal lobe.

Individuals with ASD of different ages have been reported to have cognitive flexibility difficulties in daily life (Granader et al., 2014; Leung and Zakzanis, 2014; McLean et al., 2014). Our results showed that the CFI total scores of parents of children with ASD were significantly lower than those of parents of typically developing children, suggesting that parents of autistic children had cognitive flexibility difficulties in daily life. Our results provide new evidence that cognitive flexibility may be a neurocognitive endophenotype of ASD. However, this study did not find impairment of cognitive flexibility on the task-switching task in parents of children with autism. This contrasted with the findings of Moazzen et al. (2015); Li et al. (2017), and Schmitt et al. (2019) and it is interesting that Wong et al. (2006) and McLean et al. (2014) did not report any evidence of a deficit in cognitive flexibility in parents of children with ASD using the intra-dimensional/extra-dimensional (ID/ED) shifting task and Delis–Kaplan Executive Functioning System. These inconsistencies may be due to different task paradigms. First, the broad range of cognitive abilities required to complete some tasks may interfere with the assessment of specific areas. Thus, poorer performance may be the result of executive function deficits rather than a specific impairment of cognitive flexibility (Lange et al., 2017). For example, poor performances on the WCST may not only result from cognitive flexibility, but due to various additional cognitive processes (like the high social demands, high working memory, inhibitory control, and generativity load) (Eylen et al., 2011; Albein-Urios et al., 2018). Furthermore, task difficulty is an factor in explaining the mixed findings (Geurts et al., 2009). For example, examiners may set a longer stimulus presentation time and interstimulus interval to ensure the high level of behavioral performance (Dirks et al., 2020). Another explanation is that in tests tapping executive functions explicitly providing a high degree of task instructions (like the task-switching paradigm), the examiner provides the necessary structure and organization to act as external executive control for the subject and reducing the requirement for executive functions (including cognitive flexibility) (Eylen et al., 2011). Thus, even if they do have deficit on the cognitive flexibility, they are able to compensate for these impairments with highly explicit task instructions. Finally, the use of lab-based neurocognitive tasks to measure cognitive flexibility may be limited by their limited ecological validity, which hinders their predictive value for everyday function. In any case, we did observe a dissociation between behavioral performance and self-reported impairment of cognitive flexibility in parents with ASD. Our findings showed the possibility that self-reported measures of cognitive flexibility may be more sensitive than lab-based neurocognitive measures.

Cognitive flexibility is impaired in ASD individuals (Granader et al., 2014; Leung and Zakzanis, 2014; McLean et al., 2014). In addition, there is also evidence that cognitive inflexibility is strongly associated with clinical outcomes and is important predictors of the severity of symptoms in ASD children (Kenworthy et al., 2008, 2014). The present study also showed difficulty in cognitive flexibility in parents of children with ASD and found that self-reported cognitive flexibility difficulty in parents of children with ASD was significantly negatively correlated with autistic traits, which is similar to previous findings in ASD individuals. Thus, all these findings suggested that subclinical individuals with higher autistic traits show a similar, but milder cognitive flexibility profile as individuals with ASD. However, these results should be interpreted with caution because we cannot rule out the possibility that this is not a true association. For example, CFI and BAPQ may be positively associated in part due to shared methodological effects (i.e., both are self-reported measures), since individuals may exhibit a consistent style of response (Albein-Urios et al., 2018).

The amplitude and latency of P3 were used to measure attention resource allocation and information processing speed, respectively. This study found no significant difference in P3 between the pASD and control group, indicating that both groups of subjects allocated the same amount of attention during the task. In addition, we found that the pASD group induced significantly larger N2 amplitudes, suggesting that parents of children with ASD needed to mobilize more neurocognitive resources to monitor and adapt to new changes. The N2 component is closely associated with cognitive flexibility (Kopp et al., 2020), which is supported by the findings that the N2 amplitude in the frontal lobe is significantly negatively correlated with self-reported cognitive flexibility. It has been reported that individuals with ASD induced larger N2 amplitudes under different task conditions than normally developing individuals (Faja et al., 2016; Høyland et al., 2017). These findings suggest that ASD individuals and parents of children with ASD exhibited similar atypical N2 responses. We also found a significant positive correlation between N2 amplitude in the frontal lobe and autistic traits in parents of children with ASD. We interpreted these findings as abnormal brain activity from genetic traits in first-degree relatives of ASD. In conclusion, N2 may be a neuroelectrophysiological endophenotype reflecting cognitive flexibility impairment in ASD.

The frontal lobe shows the most sustained development of any brain region (Sousa et al., 2018), which plays a vital role in executive functions involved in planning, monitoring, attention, and cognitive flexibility (D’Cruz et al., 2016; Sallet et al., 2020). Most previous neuroimaging studies of cognitive flexibility in ASD have reported atypical frontal activity (Schmitz et al., 2006; Shafritz et al., 2008; Doesburg et al., 2013; D’Cruz et al., 2016; Yeung et al., 2016; Lukito et al., 2020; May and Kana, 2020). We found that the atypical N2 responses in the parents of children with ASD were mainly in the frontal lobe. These results provide further evidence that frontal lobe dysfunction is the neural basis of cognitive flexibility impairment in individuals with autistic traits. Atypical N2 responses in the right central region in parents of children with ASD may be related to the lateralization of the brain. There is a general increase in activation in the right hemisphere and a decrease in activation in the left hemisphere with age (Rubia et al., 2006; Taylor et al., 2012). The spatial requirements of most cognitive flexibility tasks may preferentially recruit the right hemisphere as a result of development.

There are also some limitations in this study. First, we did not collect EEG data from ASD children in the early stage and only proposed hypotheses based on previous findings. If similar findings can be replicated in our own ASD cases, the research will be more systematic, and is thus planned for our future research. Second, parenting stress, anxiety, and depression level are higher in parents of children with ASD than that in parents of typically developing children (Ansari et al., 2021). Although subjects with mental illness (including anxiety and depression) were excluded, we did not take into account the possible influence of subclinical stress levels on EEG signal. In addition, future studies using lab-based neurocognitive tasks may consider a more ecological measure to provide stronger relations to everyday behaviors.

In summary, our results show that cognitive flexibility is reduced in parents of children with ASD. Impaired cognitive flexibility may be an endophenotype of ASD. In addition, self-reported measures of cognitive flexibility are sensitive. Impairment of cognitive flexibility can significantly affect the daily function and quality of life of patients with ASD and increase existing difficulties in social interaction (Albein-Urios et al., 2018). Exploring the neuropathophysiological mechanism of cognitive flexibility is helpful to further understand the neuropathophysiological mechanism of cognitive flexibility in ASD and explore effective intervention strategies to improve flexibility.

## Data Availability Statement

The original contributions presented in the study are included in the article/supplementary material, further inquiries can be directed to the corresponding authors.

## Ethics Statement

The studies involving human participants were reviewed and approved by the Medical Ethics Committee of Nanjing Brain Hospital Affiliated to Nanjing Medical University. The patients/participants provided their written informed consent to participate in this study.

## Author Contributions

XK designed the study and revised the draft. XC contributed to the design of the study, data collection and analysis, and wrote the draft of the manuscript. XWC, CL, LF, JJ, and HC contributed to the data collection. YL contributed to the data collection and analysis. ZH contributed to the manuscript revision. All authors contributed to the article and approved the submitted version.

## Conflict of Interest

The authors declare that the research was conducted in the absence of any commercial or financial relationships that could be construed as a potential conflict of interest.

## Publisher’s Note

All claims expressed in this article are solely those of the authors and do not necessarily represent those of their affiliated organizations, or those of the publisher, the editors and the reviewers. Any product that may be evaluated in this article, or claim that may be made by its manufacturer, is not guaranteed or endorsed by the publisher.
